# HLA Diversity in Transylvanian Ethnic Groups: Consequences for Hematopoietic Cell Transplantation

**DOI:** 10.3390/life14101243

**Published:** 2024-09-28

**Authors:** Lucia Dican, Mihaela Iancu, Florin Ioan Elec, Dan Burghelea, Raluca Timoce, Cristina Sorina Catana, Monica Mihaela Marta, Roxana Liana Lucaciu, Adriana Corina Hangan, Horea Vladi Matei, Luminița-Ioana Iancu Loga

**Affiliations:** 1 Department of Medical Biochemistry, Faculty of Medicine, “Iuliu-Hațieganu” University of Medicine and Pharmacy, 400012 Cluj-Napoca, Romania; lucia.dican@umfcluj.ro (L.D.); ccatana@umfcluj.ro (C.S.C.); 2 Clinical Institute of Urology and Renal Transplantation, 400000 Cluj-Napoca, Romania; ioan.elec@umfcluj.ro (F.I.E.); dan.burghelea@umfcluj.ro (D.B.); raluca.timoce@icutr.ro (R.T.); luminita.loga@icutr.ro (L.-I.I.L.); 3 Department of Medical Informatics and Biostatistics, Faculty of Medicine, “Iuliu-Hațieganu” University of Medicine and Pharmacy, 400012 Cluj-Napoca, Romania; 4 Department of Urology, Faculty of Medicine, “Iuliu-Hațieganu” University of Medicine and Pharmacy, 400012 Cluj-Napoca, Romania; 5 Department of Medical Education, Faculty of Medicine, “Iuliu-Hațieganu” University of Medicine and Pharmacy, 400012 Cluj-Napoca, Romania; mmarta@umfcluj.ro; 6 Department of Pharmaceutical Biochemistry and Clinical Laboratory, Faculty of Pharmacy, “Iuliu-Hațieganu” University of Medicine and Pharmacy, 400012 Cluj-Napoca, Romania; liana.lucaciu@umfcluj.ro; 7 Department of Inorganic Chemistry, Faculty of Pharmacy, “Iuliu-Hațieganu” University of Medicine and Pharmacy, 400012 Cluj-Napoca, Romania; adriana.hangan@umfcluj.ro; 8 Department of Cellular and Molecular Biology, Faculty of Medicine, “Iuliu-Hațieganu” University of Medicine and Pharmacy, 400012 Cluj-Napoca, Romania; hmatei@umfcluj.ro

**Keywords:** HLA allele groups frequencies, HLA haplotype frequencies ethnicity, hematopoietic stem cell, transplantation

## Abstract

The HLA profile is essential in cell and tissue transplantation, particularly in patients with autoimmune conditions and infections. Due to the extreme polymorphism in certain HLA loci, it also serves as a key tool for population genetic analysis. This study aimed to identify the allele and haplotype distributions of HLA class I (A, B, and C) and class II (DRB1) genotypes in unrelated hematopoietic stem cell donors. A retrospective analysis was conducted between 2016 and 2020 on 9832 Transylvanian volunteers, divided into Romanian and Hungarian groups based on self-reported ethnicity. Using PCR-SSO for HLA typing, significant differences were found in allele frequencies between ethnic groups. A total of 19 HLA-A, 31 HLA-B, 14 HLA-C, and 13 HLA-DRB1 distinct allele groups were identified between ethnic groups. Notably, B*18, B*51, and C*12 were more frequent in Romanians, while B*44, B*40, and C*07 were more common in Hungarians. Differences in haplotype distributions were also observed, with HLA-A*02~B*18~C*07~DRB1*11 being significantly more frequent in Romanians. Understanding these population-specific HLA profiles can improve donor matching for hematologic diseases, enhancing patient outcomes and access to life-saving hematopoietic stem cell transplantation.

## 1. Introduction

Bone marrow transplantation, or hematopoietic stem cell transplantation (HSCT), is a curative therapeutic option for blood-related disorders such as leukemia, lymphoma, and severe anemia [1]. The HLA (Human Leukocyte Antigen) profile of both the patient and the donor is crucial in cell and tissue transplantation, especially in patients with autoimmune conditions and infections. A comprehensive evaluation of the patient’s medical condition, including their HLA status, is required prior to a bone marrow transplant [2].

HLA genes are one of the most polymorphic genes in the human genome. Various subsets of peptide encoded by HLA molecules and their binding specificities are crucial for understanding the differences in the immune response between individuals [3]. HLA genes are inherited together as haplotypes [4]. Significant genetic variability was observed within the genes of the HLA system [5].

Class I and II molecules of the HLA are crucial for triggering different adaptive immune responses by presenting peptides to CD8+ or CD4+ T cells. These proteins play a crucial role in the ability of the immune system to differentiate between self and non-self cells [6].

When searching for appropriate donors for recipients on the waiting list, HLA matching is prioritized as the primary criterion. Specifically, matching at the HLA-A, -B, -C, -DRB1, -DP, and -DQ loci is considered essential for reducing the risk of complications (graft rejection and graft-versus-host disease) and maximizing the success of the transplant [7,8]. In addition to HLA matching, recent advances in the genomics of allogeneic HSCT have highlighted the importance of non-HLA genetic markers, such as killer-cell immunoglobulin-like receptors (KIRs) and other immune-related genes, in influencing transplant outcomes. These markers can modulate post-transplant immune responses, affecting graft success and patient recovery. Understanding the complex interplay between HLA and non-HLA markers, as well as the impact of HLA downregulation in malignancies, is critical for optimizing donor selection and improving HSCT outcomes [9,10,11,12].

Haplotypes represent combinations of alleles inherited together on the same chromosome from one parent. Matching at the haplotype level ensures compatibility across multiple HLA loci simultaneously, providing a more comprehensive assessment of compatibility compared to allele-level matching, which considers each locus independently [13]. A patient’s siblings frequently have the best HLA match. If a suitable sibling donor is unavailable, the search for unrelated donors is conducted in national and international registries. A complete HLA match is desired; however, partial matches are acceptable if the mismatch is below tolerable levels [14]. National Registry of Hematopoietic Stem Cells Voluntary Donors (NRHSCVD) comprises 0.33% of the nation’s population. Any increase in registrations enhances the likelihood of saving patients with blood cancer or other serious hematological conditions. Internationally, 49% of blood cancer patients in need of a hematopoietic stem cell transplant may find a suitable unrelated donor in hematopoietic stem cell donor registries worldwide. Only 67% of Romanian patients requiring a hematopoietic stem cell transplant from an unrelated donor can find a genetically suitable donor match. A total of 96% of donors compatible with Romanian patients who underwent transplantation were sourced from international registries, while only 4% were found in the national registry. Only 20% of patients from an ethnic minority can find a matching donor. To increase the chances of finding a suitable donor for the 33% of patients without matches in international registries, more than 5% of the Romanian population needs to register in the NRHSCVD, which is linked to registries of hematopoietic stem cell donors in 54 countries worldwide, totaling over 38 million volunteer donors. A total of 8% of patients worldwide who need a transplant do not have a matching donor. NRHSCVD reported that over 320,000 prospective donors listed in international databases had already donated hematopoietic stem cells to unrelated patients [15]. According to the 2021 Romanian census, out of the total 19,053,815 population of Romania, 1,002,151 are Hungarian ethnics, most of them located in the multi-ethnic region of Transylvania, where they are the main ethnic minority [16]. Transylvania is a multi-ethnic region in Romania with diverse ethnicities (Romanians, Hungarians, and German Saxons). Romanian native language is an Eastern Romance language derived from Latin. Hungarians are speakers of the Hungarian language and are members of the Uralic language family [17]. The Hungarian population in Romania constitutes the largest Hungarian diaspora outside of Hungary. After the Romanian Revolution, there was a significant influx of migrants for many decades. There is limited research on Romanian unrelated donors according to HLA haplotypes. However, a single-center study compared new histocompatibility predictive algorithms with donor matching according to HLA [18]. HLA genotyping in the Romanian population focused on the most common alleles in living donors in an attempt to design an HLA distribution map to facilitate the finding of compatible donor matches [19]. Another center study showed that Romanians share genetic traits with other Europeans [20], while potential genetic differences across the Romanian regions were revealed, but without providing a comprehensive comparison between Romanian and Hungarian ethnics, although race and ethnicity continue to be important factors in the identification of unrelated donors according to HLA. Prior studies have investigated the HLA variety of Romanians based on area [19,20,21]. Different ethnic groups and individuals of the same ethnic group living in different regions have variable HLA allele and haplotype distributions [22]. When ethnicity self-identification information is unavailable, many methods have been suggested to categorize populations by name [23]. HLA depends on race/ethnicity for identifying unrelated donors.

In bone marrow transplantation, genetic polymorphism is assessed through DNA typing to determine compatibility between the donor and recipient at the HLA loci.

These DNA typing methods enable healthcare professionals to accurately assess the genetic compatibility between donors and recipients in bone marrow transplantation. Advancements in genomic technologies, particularly next-generation sequencing, have improved the precision of HLA typing, facilitating more accurate donor–recipient matching and reducing complications such as graft-versus-host disease (GvHD). This enhanced matching is critical, particularly in ethnically diverse populations like those in Transylvania, where minor genetic differences can significantly impact transplant outcomes. By matching HLA alleles at multiple loci, the risk of graft rejection and graft-versus-host disease (GVHD) can be minimized, leading to improved outcomes for transplant recipients [24].

The objectives of this study were as follows: [i] to describe the distributions of the HLA-A, -B, -C, and –DRB1 alleles frequencies stratified by ethnicity group, [ii] to identify the multi-locus haplotype frequencies stratified by ethnicity group, and [iii] to compare the most common haplotypes in the two studied ethnic groups.

## 2. Materials and Methods

### 2.1. Population Dataset

The ethics council of the Clinical Institute of Urology and Renal Transplantation Cluj-Napoca approved the research (no. 01/27 January 2023). Following guidelines, patients were included after receiving written informed consent for sample collection and analysis. The current study included the samples of NRHSCVD who were registered at the Laboratory of Clinical Immunology, Clinical Institute of Urology, and Renal Transplantation Cluj-Napoca.

A total of 9832 unrelated stem cell donors from two ethnic groups were enrolled in this retrospective study between 2016 and 2020. The volunteers were aged 18–50 years old. The individuals were divided into two groups: the Romanian majority group (RO = 8333) and the Hungarian minority group (HUN = 1499). Romanian and Hungarian subjects were from Transylvania. Individuals were grouped by ethnicity based on their self-declared ethnicity at the time of donor registration.

Blood samples were collected, and DNA was extracted using an innuPREP Blood DNA Mini kit IPC16 (Analytik Jena AG, Berlin, Germany) according to the manufacturer’s protocol. DNA sample concentrations were quantified with a NanoPhotometer and adjusted to a concentration of 10–30 ng/μL.

HLA allele groups were molecularly typed at low/intermediate resolution for the HLA class I and HLA class II alleles based upon the Polymerase Chain Reaction Sequence-Specific Oligomer Probe Hybridization (PCR-SSO) using commercial reagents and following the instructions of the manufacturer (BAG Health Care GmbH. Germany). The analysis of HLA data was performed using HISTO MATCH Software (V4.X-03/2020). When necessary, ambiguous HLA alleles were retested via the Polymerase Chain Reaction Sequence-Specific Primer (PCR-SSP) using the HLA A-B-DR SSP Combi Tray [CareDx. Stockholm, Sweden] according to the manufacturer’s instructions. The results were processed with the Helmberg SCORE 5.00.41T software.

### 2.2. Statistical Analysis

Qualitative variables (such as sex and alleles of HLA-A, HLA-B, HLA-C, and HLA-DRB1) were described with absolute and relative frequencies. The chi-square test was used to compare distributions of sex between studied ethnic groups. The relative frequencies (AF) of HLA-A, HLA-B, HLA-C, and HLA-DRB1 allelic groups were calculated by direct counting using R v. 4.3.2. We computed the maximum likelihood estimates of the HLA haplotype frequencies (HF) and the posterior probabilities of the pairs of haplotypes for each subject using the expectation-maximization (EM) algorithm from haplo.stats R package [25]. The estimated haplotypes with a relative frequency (HF, %) lower than 0.5% were not presented. In order to identify significant differences in the most common multi-locus haplotype frequencies between two ethnic groups, we performed the Score test [26].

We tested deviations from Hardy–Weinberg equilibrium (HWE) for each locus by chi-square test from the “gap” R package [27], and Bonferroni-adjusted *p*-values for multiple testing were reported. For each locus pair, we tested the linkage disequilibrium [LD] by the following measures: the weighted average of normalized disequilibrium value [D’], the correlation coefficient between alleles at two loci [W_n_], and the conditional asymmetric linkage disequilibrium measurements [W_Locus1_/_Locus2_ and W_Locus2_/_Locus1_] using the” pould” R package [28]. The coefficients W_Locus1_/_Locus2_ and W_Locus2_/_Locus1_ were extensions of the W_n_ for highly polymorphic loci, the measure W_Locus1_/_Locus2_ quantifying the variation of alleles at Locus 1 on any of the haplotypes conditioned by alleles at Locus2 while the W_Locus2_/_Locus1_ measured the variation of alleles at Locus2 on any of the haplotypes conditioned by alleles at Locus 1.

For all two-sided statistical tests, we used a significance level of alpha = 0.05. A significant result is achieved when the estimated significance level *p*-value (unadjusted and corrected) < 0.05.

## 3. Results

### 3.1. HWE Equilibrium in Living Donors’ Sample

There was no significant deviation from Hardy–Weinberg equilibrium at the HLA-A locus (*p*-corrected = 1.0000), HLA-C locus (*p*-corrected = 0.9416), and DRB1 locus (*p*-corrected = 1.0000), while the HLA-B locus showed a significant departure from Hardy–Weinberg equilibrium (*p*-corrected < 0.00001) among unrelated stem cell donors.

### 3.2. Estimates of Linkage Disequilibrium between HLA-A, HLA-B, HLA-C, and DRB1 Loci in Unrelated Stem Cell Donors’ Sample and Studied Groups

Linkage disequilibrium (LD) and asymmetric linkage disequilibrium (ALD) measures between two-locus haplotypes in all unrelated blood donor volunteers’ samples and studied groups are presented in Table 1.

Within each of the studied groups, the values of D’ and W_n_ were comparable for all locus pairs (e.g., A~C vs. A~DRB1), while a strong linkage disequilibrium was noticed between HLA-B and HLA-C loci (D’ = 0.80, W_n_ = 0.67 for Romanian ethnic group, D’ = 0.81, W_n_ = 0.67 for Hungarian group).

The highest ALD values were found between HLA-B and HLA-C in both studied groups (RO group: W_B/C_ = 0.56, W_C/B_ = 0.74 and W_B/C_ = 0.56, W_C/B_ = 0.75 for HUN group, respectively) while the lower ALD values were found between more distant loci (e.g., HLA-A and DRB1 and between HLA-A and C) (Table 1).

In all living donors’ samples, the sex ratio was 1.84 male/female [6403 (65.12%) males vs. 3429 (34.88%) females], with no significant difference in sex distributions between the two ethnic studied groups (chi-square test, *p* = 0.0536).

### 3.3. Allelic Groups Frequency for HLA-A, HLA-B, HLA-C, and HLA-DRB1

The allele group frequencies of studied antigens HLA-A, -B, -C, and -DRB1 among unrelated stem cell donors are summarized in Table 2, Table 3 and Table 4. A total of 19 distinct allele groups were detected in all samples of donors at the HLA-A locus. The most frequent allele groups were A*02 (27.07%), succeeded by A*01 (13.98%), A*24 (11.80%), A*03 (11.36%), and A*11 (7.21%). When stratifying by ethnicity group, the most common allele group for the HLA-A locus remained the same (Table 2). In addition, we noticed a difference in the frequency of the allele groups A*01, A*25, and A*36 between ethnic subgroups with a tendency toward statistical significance (*p* < 0.05, p_BH_ = 0.087). In donors’ samples, a total of 31 different allele groups were detected at the HLA-B locus (Table 3), with the prevalent allele group including B*35 (14.71%), followed by B*18 (10.73%), B*51 (9.77%), B*44 (9.29%), and B*08 (8.37%).

We found a significant difference in the frequency of allele B*18 (p_BH_ = 0.0039, 95% CI for difference in frequency (%): [0.9; 3.2]), B*51 (p_BH_ < 0.0001, 95% CI: [1.8, 3.9]), and B*47 (p_BH_ = 0.0035, 95% CI: [0.3, 0.7]), which were significantly higher in the Romanian than in the Hungarian group, while B*44 (p_BH_ = 0.0280, 95% CI: [−2.7, −0.04]), B*40 (p_BH_ = 0.0028, 95% CI: [−2.4, −0.6]), B*57 (p_BH_ = 0.0061, 95% CI: [−1.4, −0.2]), and B*48 (p_BH_ < 0.0001, 95% CI: [−0.8, −0.2]) were significantly lower in the Romanian than in the Hungarian group (Table 3).

A total of 14 distinct HLA-C allele groups were detected among all unrelated blood donor volunteers, with C*07 as the most prevalent, having a relative frequency of 24.89%. Additional common allele groups were the following: C*04 (16.23%), C*12 (14.10%), C*06 (8.44%), C*02 (7.72%), and C*03 (6.63%). A total of 13 distinct HLA-DRB1 alleles were identified in donors (Table 4), with DRB1*11 as the most common allele having a relative frequency of 19.21%. Other prevalent alleles included the following: DRB1*13 (11.06%), DRB1*03 (10.90%), DRB1*16 (10.89%), DRB1*07 (10.76%), and DRB1*01 (9.79%).

We found a significant difference in the frequency of C*12 (p_BH_ = 0.0091), C*01 (p_BH_ = 0.0035), and C*14 (p_BH_ = 0.0091), which were significantly higher in Romanian than in Hungarian group (95% CI for difference in frequency of C*12 allele: [0.8, 3.4], 95% CI for difference in frequency of C*01 allele: 95% CI: [0.8, 2.4], 95% CI for difference in frequency of C*14 allele: [0.4, 1.2]). The alleles C*07 (p_BH_ = 0.0146, 95% CI: [−4.1, −0.7]), C*05 (p_BH_ = 0.0280, 95% CI: [−1.7, −0.1]), and C*08 (p_BH_ = 0.0014, 95% CI: [−1.9, −0.5]) were significantly lower in the Romanian than in the Hungarian group (Table 4). Also, we found a significant difference in the frequency of alleles DRB1*11, DRB1*16, and DRAB1*14 between ethnic groups (Table 4).

**Figure 1:**
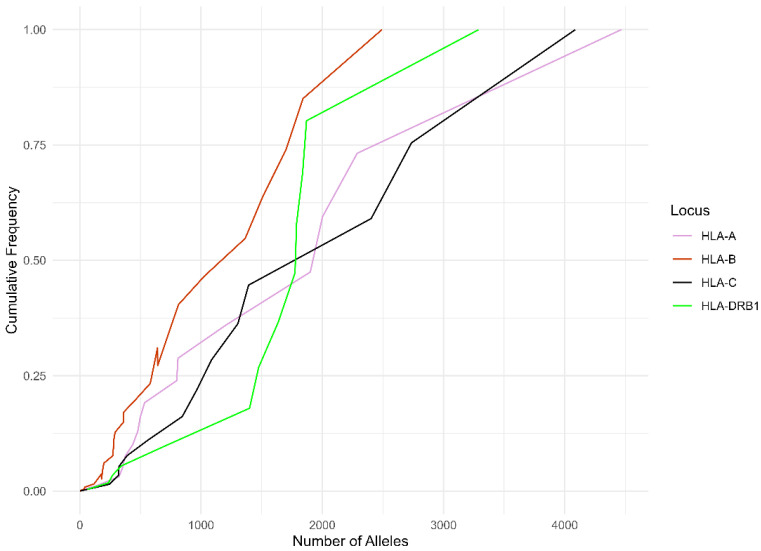
Cumulative frequency of the alleles by each HLA in the Romanian group.

**Figure 2:**
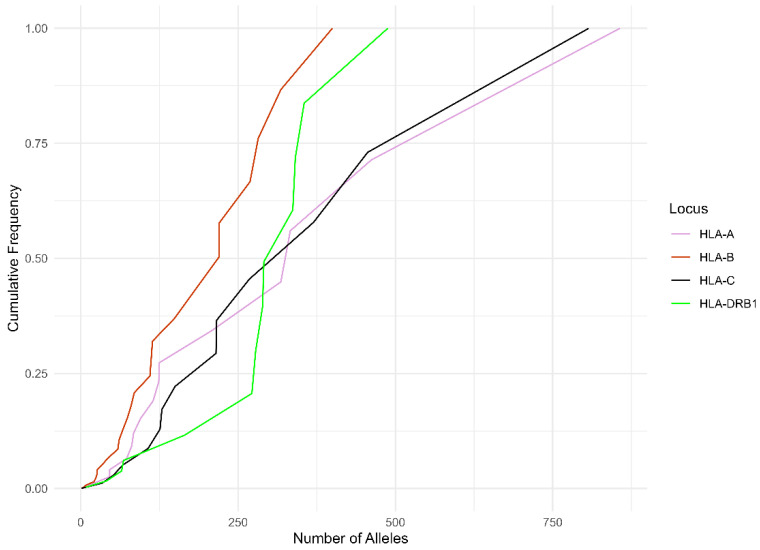
Cumulative frequency of the alleles by each HLA in the Hungarian group.

The HLA-B has the highest allelic diversity in both studied groups (Figure 1 and Figure 2). In the Romanian group, the cumulative frequency of each locus for the top five most frequent allele groups was 76.07% for the HLA-A, 53.45% for the HLA-B, 71.53% for the HLA-C, and 63.36% for the HLA-DRB1, while in the Hungarian group, we noticed that the cumulative frequencies of each locus were 72.66% for the HLA-A, 49.67% for the HLA-B, 70.58% for the HLA-C, and 59.80% for the HLA-DRB1.

Figure 1 and Figure 2 illustrate the alleles that provide the highest level of diversity within HLA Class I and Class II. HLA-A and -C had the greatest cumulative frequency, while HLA-B exhibited the lowest cumulative frequency. In addition, the HLA-A and -C curves reached a cumulative frequency of 100% before the HLA-B curve and HLA-DRB1 curve. This result validates that HLA-A and -C exhibits the least variation, whereas HLA-B has the greatest allelic variety.

We found no significant difference in the frequencies of observed HLA-A genotypes (*p* = 0.4214, homozygous: 639 [7.7%] in the RO group vs. 106 [7.1%] in the HUN group) between the two ethnic groups. Similar results were obtained for HLA-B genotypes (*p* = 0.7382, homozygous: 1146 [13.8%] in the RO group vs. 211 [14.1%] in the HUN group), HLA-C genotypes (*p* = 0.2474, homozygous: 1216 [14.6%] in the RO group vs. 236 [15.7%] in the HUN group), and HLA-DRB1 genotypes (*p* = 0.4047, homozygous: 974 [11.7%] in the RO group vs. 164 [10.9%] in the HUN group).

### 3.4. Estimated Two-, Three-, and Four-Locus Haplotype Frequency between Studied Groups

The most frequent HLA-A-B, HLA-A-C, HLA-A-DRB1, HLA-B-C, HLA-B-DRB1, HLA-C-DRB1, HLA-A-B-C, HLA-A-B-DRB1, HLA-A-C-DRB1, HLA-B-C-DRB1, and HLA-A-B-C-DRB1 haplotypes are summarized in Supplementary Tables S1–S3. In total, 389 HLA-A-B, 208 HLA-A-C, 212 HLA-A-DRB1, 217 HLA-B-C, 321 HLA-B-DRB1, 166 HLA-C-DRB1, 1013 HLA-A-B-C, 932 HLA-B-C-DRB1, 1341 HLA-A-C-DRB1, and 2,883 HLA-A-B-C-DRB1 distinct haplotypes were identified among unrelated stem cell donors. We found that the most frequent two-locus HLA haplotypes, with a relative haplotype frequency > 4% (Table 5), were as follows: A*01~B*08 (5.9%), A*02~B*18 (4.1%), A*02~C*07 (7.6%), A*01~C*07 (7.1%), A*02~DRB1*11 (6.2%), A*01~DRB1*03 (5.1%), A*02~DRB1*16 (4.1%), B*35~C*04 (12.9%), B*08~C*07 (8.2%), B*18~C*07 (5.7%), B*01~C*07 (5.2%), B*08~DRB1*03 (6.8%), B*18~DRB1*11 (5.7%), C*07~DRB1*03 (7.4%), and C*07~DRB1*11 (5.6%).

A total of 375 HLA-A-B, 202 HLA-A-C, 205 HLA-A-DRB1, 203 HLA-B-C, 309 HLA-B-DRB1, 164 HLA-C-DRB1, 363 HLA-A-B-C, 427 HLA-A-B-DRB1, 358 HLA-B-C-DRB1, 357 HLA-A-C-DRB1, and 632 HLA-A-B-C-DRB1 distinct haplotypes were detected among Romanian donors subjects. A number of 272 HLA-A-B, 167 HLA-A-C, 172 HLA-A-DRB1, 132 HLA-B-C, 250 HLA-B-DRB1, 144 HLA-C-DRB1, 363 HLA-A-B-C, 427 HLA-A-B-DRB1, 358 HLA-B-C-DRB1, 357 HLA-A-C-DRB1, and 632 HLA-A-B-C-DRB1 distinct haplotypes were detected among Hungarian donors subjects.

The most common two-locus HLA haplotypes, with a relative haplotype frequency surpassing 3% [Supplementary Tables S1–S3] in Romanian and Hungarian groups, were as follows: A*01~B*08 (5.7% vs. 6.9%, *p* = 0.01644), A*02~B*18 (4.3% vs. 3.0%, *p* = 0.00168), A*02~C*07 (7.7% vs. 7.2%, *p* = 0.82983), A*01~C*07 (6.9% vs. 8.0%, *p* = 0.02522), A*03~C*07 (3.0% vs. 4.1%, *p* = 0.05235), B*08~DRB1*03 (6.7% vs. 7.5%, *p* = 0.1131), B*18~DRB1*11 (6.0% vs. 4.2%, *p* = 0.00009), B*35~DRB1*11 (3.9% vs. 3.2%, *p* = 0.03500), B*35~DRB1*01 (3.3% vs. 2.9%, *p* = 0.15017), C*07~DRB1*03 (7.3% vs. 8.2%, *p* = 0.07192), C*07~DRB1*11 (5.9% vs. 5.3%, *p* = 0.2390), C*06~DRB1*07 (4.1% vs. 4.3%, *p* = 0.70461), C*04~DRB1*11 (3.9% vs. 3.3%, *p* = 0.08844), C*04~DRB1*01 (3.6% vs. 2.8%, *p* = 0.04552), B*35~C*04 (13.2% vs. 11.8%, *p* = 0.0344), B*08~C*07 (8.0% vs. 9.3%, *p* = 0.01855), B*18~C*07 (5.9% vs. 4.7%, *p* = 0.00943), B*07~C*07 (5.0% vs. 6.5%, *p* = 0.00098), B*18~C*12 (3.9% vs. 3.2%, *p* = 0.05156), A*02~DRB1*11 (6.5% vs. 4.8%, *p* = 0.00207), A*01~DRB1*03 (4.9% vs. 5.8%, *p* = 0.04229), and A*02~DRB1*16 (4.3% vs. 3.6%, *p* = 0.06539).

The haplotype analysis performed among unrelated stem cell donors highlighted as the most common three-locus haplotypes, with a relative haplotype frequency exceeding 3%, as follows: A*01~B*08~C*07 (5.9%), A*02~B*18~C*07 (3.4%), A*01~B*08~DRB1*12 (4.9%), B*08~C*07~DRB1*12 (6.8%), B*18~C*07~DRB1*04 (4.1%), B*35~C*04~DRB1*04 (3.4%), B*35~C*04~DRB1*01 (3.1%), A*01~C*07~DRB1*12 (4.9%), and A*02~C*07~DRB1*04 (3.1%) (Supplementary Tables S4 and S5).

The most common three-locus haplotypes observed among Romanian donors subjects and Hungarian donors, with a relative haplotype frequency exceeding 3% (Supplementary Tables S4 and S5), were as follows: A*01~B*08~C*07 (5.7% versus 6.8%, *p* = 0.01621), A*02~B*18~C*07 (3.6% versus 2.7%, *p* = 0.0035), A*01~B*08~DRB1*03 (4.8% versus 5.6%, *p* = 0.12059), A*02~B*18~DRB1*11 (3.2% versus 2%, *p* = 0.00006), B*08~C*07~DRB1*03 (6.6% versus 7.4%, *p* = 0.10395), B*18~C*07~DRB1*11 (4.3% versus 3.1%, *p* = 0.00128), B*35~C*04~DRB1*11 (3.5% versus 2.9%, *p* = 0.06605), B*35~C*04~DRB1*01 (3.2% versus 2.7%, *p* = 0.13018), A*01~C*07~DRB1*03 (4.8% versus 5.6%, *p* = 0.06287), and A*02~C*07~DRB1*11 (3.2% versus 2.0%, *p* = 0.01242).

The most frequent four-locus haplotypes among all stem cell donors, with a relative haplotype frequency > 1%, were as follows: A*01~B*08~C*07~DRB1*12 (4.9%), A*02~B*18~C*07~DRB1*04 (2.8%), and A*03~B*35~C*04~DRB1*01 (1.3%) (Table 5).

The most common four-locus haplotypes among Romanian donors subjects and Hungarian donors groups, a relative haplotype frequency exceeding 1% (Table 5) were as follows: A*01~B*08~C*07~DRB1*03 (4.8% versus 5.6%, *p* = 0.1573), A*02~B*18~C*07~DRB1*11 (3.0% versus 1.9%, *p* = 0.00086), and A*03~B*35~C*04~DRB1*01 (1.4% versus 1.1%, *p* = 0.23272).

## 4. Discussion

This study presents the first analysis of HLA-A, -B, -C, and -DRB1 allele groups and haplotype (two, three, and four loci) frequencies in two Transylvanian ethnic groups. Previous studies conducted in the Romanian population [20,21] used a smaller number of samples compared to our study, which included 9832 people from Transylvania, Romania.

The five HLA-A allele groups (A*02, A*01, A*24, A*03, and A*11) with frequencies above 5.0% accounted for 64.21% of the allelic variation detected at this locus. HLA-A*02 was also the most frequent at the HLA-A locus in another study conducted in the Hungarian population [29]. A recent study found that HLA-A*02 alongside HLA-A*24 was prevalent worldwide, which correlates with our findings [30]. HLA-A*02 and HLA-A*01 were jointly the most common HLA-A allele groups in the mixed populations [31].

The frequency of only six HLA-B allele groups (B*35, B*18, B*51, B*44, B*08, and B*07), representing 58.21% of the HLA-B in the Romanian group, was greater than 5%.

As far as HLA-B was concerned, HLA-B*35 was the most common allele group in both our ethnic groups (14.96% and 13.34%, respectively), similar to a previous study reporting HLA-B*35 as the most common allele group in a Romanian population (13.9%) [17,18,19]. HLA-B*35 was also identified as the most common allele in Turks [32], Serbians [33], and Italians [34].

HLA–B*44 was the second most polymorphic in the Hungarian group, while HLA-B*18 was the second most polymorphic in the Romanian group. These findings have implications for finding a suitable donor for patients with B*44. The second most frequent HLA-B allele group was HLA-B*18, which is highly prevalent in Caucasians and only rarely seen in Black populations. Similar frequencies were observed in Bulgarians and Macedonians [35]. In Western European populations, the most common HLA-B allele groups were B*08, B*07, B*44, B*35, and B*40 [36].

Only eight allele groups of HLA-C (C*07, C*04, C*12, C*06, C*02, C*03, C*01, and C*15) had a frequency above 5%. These allele groups account for 58.29% of all HLA-C in our two studied groups. HLA-C*07 was also found to be the most common allele group in Europe [37].

Eight HLA-DRB1 (DRB1*11, DRB1*13, DRB1*03, DRB1*16, DRB1*07, DRB1*01, DRB1*15, and DRB1*04) had a frequency greater than 5%, accounting for 90.12% of all HLA-DRB1 in both our groups. The HLA-DRB1*11 and DRB1*03 are prevalent in the central and eastern parts of Europe, as reported in earlier studies [38]. Previous research showed that HLA-DRB1*11 allele groups were common in both Romanians and Hungarians [20,21,28].

There were significant differences in allele group frequencies between population samples, B*18, B*51, B*47, C*12, C*01, and C*14, DRB1*11, and DRB1*16 were significantly higher in Romanian than in Hungarian group, while B*44, B*40, B*57, B*48, C*07, C*05, C*08, DRB1*14 were significantly lower in Romanian than in Hungarian group [29].

We found here that the Transylvanian population presents the highest frequency levels of homozygosity at HLA-C, followed by HLA-B, HLA-DRB1, and HLA-A. HLA homozygosity offers benefits in allogeneic HSCT, including fewer alleles to match and equivalent outcomes to haplotype identical transplants. The HLA region has one of the highest levels of LD in the human genome. HLA-B and HLA-C alleles exhibit very strong LD, as measured in the control samples. Our results are consistent with other studies that showed similar genetic frequency differences between northern and southern East European populations [33].

It was estimated that among two-loci haplotypes with frequencies higher than 1%, 48,52% of A~B haplotypes, 64.71% of A~C haplotypes, 56.24% of A~-DRB1 haplotypes, 55.33% of B~DRB1 haplotypes, 62% of C~DRB1* and 78.57% of B~C haplotypes had significant linkage disequilibrium. B*35~C*04 is found at a frequency higher than 10% in all populations studied [29].

A large number of HLA-A~B~-C haplotypes were observed in each of the Transylvanian populations described in this study. Examples of three-locus A~B~-C haplotypes in the RO group vs. HUN group (frequency > 2%) were A*01~B*08~C*07 (5.7% versus 6.8%, *p* = 0.01621) and A*02~B*18~C*07 (3.6% versus 2.7%, *p* = 0.0035).

The predominant HLA-A~B~C~DRB1 haplotype observed in this study is A*01~B*08~C*07~DRB1*03, which is widely observed in Europeans. The second most prevalent haplotype is A*02~B*18~C*07~DRB1*11 (3.0% RO versus 1.9% HUN, *p* = 0.00086). A*02~B*18~C*07~DRB1*11 has been found in other populations like Greeks [39], Germans [40], Italians [41], French [42], Macedonians [43], Albanians [44], and Sicilians [45].

HWE was found in HLA-A, -C, and HLA-DRB1 loci but not HLA-B [46]. Our investigation of the donor population found departures from HWE proportions in HLA-B for both ethnic groups. The results of our study showed that there are usually minor changes in HLA allele and haplotype across various Romanian and Hungarian. The strong linkage disequilibrium seen between B-C locus pairings in our research indicates minimal recombination changes between alleles from these loci in the Transylvanian population; therefore, the findings presented in this study provide the first thorough examination of four loci in the two ethnic populations from Transylvania. The allele frequency, haplotype frequency, and linkage disequilibrium statistics acquired in this research are useful for facilitating the search for unrelated matched donors and may assist in national donor recruitment strategy.

The expansion of non-HLA genomic research is a critical future direction in the field of hematopoietic stem cell transplantation (HSCT). With the growing recognition of the role that non-HLA markers, particularly killer-cell immunoglobulin-like receptors (KIRs), play in influencing transplant outcomes, future research should prioritize understanding how these and other immune-related genes affect transplant success and patient recovery.

### Study Limitations

Considering that the haplotype frequencies described in the current study are estimates based on the allele frequencies, information about the ancestors of each donor and future studies are required to identify and confirm the true inherited haplotypes. The results of the present study are specific to the Transylvanian population, taking into account that the observed relative frequency of Hungarian donors in the current study was 15.25%, and it may not be stable for the entire state. In addition, HLA-B loci presented deviation from Hardy–Weinberg equilibrium, so it may suggest the influence of selectivity in our population. Further studies are necessary to explore the genetic mechanisms in the target population.

## 5. Conclusions

Our analysis confirmed the heterogeneity of the Transylvanian population and the genetic similarity of nearby European population groups as far as the diversity of HLA haplotypes and polymorphisms is concerned. Such a preliminary study facilitates the recruitment of hematopoietic stem cell donors for subsequent genetic testing prior to transplantation. Analysis of HLA class I as well as II polymorphism in the Transylvania population reveals that prevalent alleles in HLA-A, -B, -C, and -DRB1 loci are similar, with small differences across RO and HUN groups. HLA haplotype frequencies in different ethnic groups help design a better plan for donor centers in different provinces of a country, predict donor size in the registry, and find suitable donors for hematopoietic stem cell transplant patients. While our study confirms that the populations are genetically very similar, we acknowledge that genetic variations upon population segregation can still occur. These variations may arise due to historical migrations, geographic isolation, or genetic drift, particularly in multi-ethnic regions like Transylvania. Such subtle differences, as seen in the distinct HLA allele and haplotype distributions between Romanians and Hungarians, may have important implications for donor compatibility in hematopoietic stem cell transplantation. Further research focusing on these genetic variations could provide a deeper understanding of their impact on transplant outcomes. In summary, understanding the HLA gene profile of an ethnic population in a geographic area is critical for guiding the search for compatible stem cell donors in hematologic diseases. By prioritizing donor searches within specific ethnic populations, healthcare providers can improve matching, enhance patient satisfaction, and increase access to life-saving treatments for patients in need of HSCT. In Romania, where the national registry is smaller and the population is ethnically diverse, matching can be particularly challenging, especially for minority groups such as Hungarians in Transylvania.

## Figures and Tables

**Table 1:** Estimates of linkage disequilibrium between all pairs of studied loci in all samples and studied groups.

			Ethnic Groups	
Loc1~Loc2	Measures	Donors Sample (N = 9832)	RO (N_1_ = 8333)	HUN (N_2_ = 1499)
	D’	0.3635	0.3679	0.3775
A~B	W_n_	0.2674	0.2694	0.2901
	W_Loc2/Loc1_	0.2674	0.2675	0.2894
	W_Loc1/Loc2_	0.3148	0.3168	0.3303
	D’	0.2759	0.2795	0.3131
	W_n_	0.2461	0.2469	0.2709
A~C	W_Loc2/Loc1_	0.2524	0.2529	0.2784
	W_Loc1/Loc2_	0.1988	0.2002	0.2210
	D’	0.2169	0.2236	0.2418
	W_n_	0.1621	0.1649	0.1772
A~DRB1	W_Loc2/Loc1_	0.1921	0.1952	0.2030
	W_Loc1/Loc2_	0.1787	0.1805	0.1961
	D’	0.7991	0.7987	0.8076
B~C	W_n_	0.6659	0.6666	0.6670
	W_Loc2/Loc1_	0.7400	0.7390	0.7509
	W_Loc1/Loc2_	0.5598	0.5611	0.5604
	D’	0.4238	0.4261	0.4434
B~DRB1	W_n_	0.3439	0.3456	0.3575
	W_Loc2/Loc1_	0.3973	0.3996	0.4067
	W_Loc1/Loc2_	0.2987	0.2992	0.3122
C~DRB1	D’	0.3259	0.3252	0.3529
	W_n_	0.2201	0.2130	0.2414
	W_Loc2/Loc1_	0.2520	0.2519	0.2728
	W_Loc1/Loc2_	0.2729	0.2729	0.2901

Loc1 = Locus 1; Loc2 = Locus 2; D’ = weighted average of normalized disequilibrium (D) values; Wn = correlation coefficient between alleles at two loci; W_Loc1/Loc2_ = variation at Locus 1 conditioned by the variation at the second locus; W_Loc2/Loc1_ = variation at Locus 2 conditioned by the variation the first locus.

**Table 2:** Distribution of HLA-A allele groups frequency in sample of unrelated stem cell donors from Transylvania stratified by ethnicity.

	All Donors (N = 9832)		Ethnic Groups					
			RO N_1_ = 8333		HUN N_2_ = 1499		*p*-Value	p_BH_
HLA-A Allele Group	n	AF (%)	n	AF (%)	n	AF (%)		
*02	5324	27.07	4467	26.8	857	28.59	0.0432 *	0.1296
*01	2750	13.98	2288	13.73	462	15.41	0.0145 *	0.087
*24	2320	11.8	2002	12.01	318	10.61	0.0281 *	0.1210
*03	2234	11.36	1901	11.41	333	11.11	0.6348	0.6721
*11	1418	7.21	1210	7.26	208	6.94	0.5299	0.6721
*32	935	4.75	810	4.86	125	4.17	0.1018	0.2291
*26	923	4.69	799	4.79	124	4.14	0.1168	0.2336
*68	629	3.2	534	3.2	95	3.17	0.9193	0.9193
*25	613	3.12	498	2.99	115	3.84	0.0139 *	0.0870
*23	559	2.84	478	2.87	81	2.7	0.6139	0.6721
*30	521	2.65	437	2.62	84	2.8	0.5726	0.6721
*31	445	2.26	372	2.23	73	2.43	0.4917	0.6721
*33	401	2.04	355	2.13	46	1.53	0.0336 *	0.1210
*29	369	1.88	323	1.94	46	1.53	0.1337	0.2407
*66	157	0.8	138	0.83	19	0.63	0.2712	0.4438
*69	35	0.18	28	0.17	7	0.23	0.4336	0.6504
*74	20	0.1	20	0.12	ND	ND		
*34	8	0.04	5	0.03	3	0.1	0.0799	0.2055
*36	3	0.02	1	0.01	2	0.07	0.0132 *	0.0870
19 alleles			19 alleles		18 alleles			

HLA = human leucocyte antigen; n = number of alleles; number of subjects = N (N_1_ = 8333, N_2_ = 1499); number of alleles = 2*N_i_; i = 1, 2; AF [%] = relative frequency of allele [%]; ND = not detected; * significant result: *p* < 0.05; *p*-values were obtained from two-proportions z-test; p_BH_ = corrected *p*-values using the Benjamini–Hochberg [BH] method.

**Table 3:** Distribution of HLA-B allele frequencies in unrelated stem cell donor volunteers sample stratified by ethnicity.

			Ethnic Groups					
	All Donors (N = 9832)		RO (N_1_ = 8333)		HUN (N_2_ = 1499)			
HLA-B Allele	n	AF	n	AF	n	AF	*p*-Value	p_BH_
*35	2893	14.71	2493	14.96	400	13.34	0.0214 *	0.0599
*18	2110	10.73	1841	11.05	269	8.97	0.0007 *	0.0039 *
*51	1921	9.77	1701	10.21	220	7.34	<0.0001 *	<0.0001 *
*44	1827	9.29	1509	9.05	318	10.61	0.0070 *	0.0280 *
*08	1646	8.37	1364	8.18	282	9.41	0.0261 *	0.0664
*07	1247	6.34	1027	6.16	220	7.34	0.0150 *	0.0525
*27	963	4.9	814	4.88	149	4.97	0.8412	0.9229
*40	931	4.73	750	4.5	181	6.04	0.0003 *	0.0028 *
*38	756	3.84	642	3.85	114	3.8	0.8965	0.9229
*15	753	3.83	641	3.85	112	3.74	0.7720	0.9229
*13	691	3.51	581	3.49	110	3.67	0.6164	0.8901
*39	541	2.75	461	2.77	80	2.67	0.7634	0.9229
*14	444	2.26	359	2.15	85	2.84	0.0208 *	0.0599
*52	428	2.18	361	2.17	67	2.23	0.8123	0.9229
*49	350	1.78	291	1.75	59	1.97	0.3976	0.6549
*57	346	1.76	272	1.63	74	2.47	0.0013 *	0.0061 *
*41	338	1.72	277	1.66	61	2.03	0.1484	0.3197
*55	323	1.64	280	1.68	43	1.43	0.3297	0.5770
*37	232	1.18	198	1.19	34	1.13	0.8011	0.9229
*56	214	1.09	190	1.14	24	0.8	0.0990	0.2310
*50	205	1.04	179	1.07	26	0.87	0.3047	0.5770
*58	204	1.04	178	1.07	26	0.87	0.3178	0.5770
*47	123	0.63	118	0.71	5	0.17	0.0005 *	0.0035 *
*48	52	0.26	31	0.19	21	0.7	<0.0001 *	<0.0001 *
*45	47	0.24	41	0.25	6	0.2	0.6358	0.8901
*53	47	0.24	38	0.23	9	0.3	0.4561	0.7095
*73	14	0.07	12	0.07	2	0.07	0.9203	0.9229
*42	5	0.03	5	0.03	ND	ND		
*46	6	0.03	5	0.03	1	0.03	0.9229	0.9229
*54	6	0.03	6	0.04	ND	ND		
*81	1	0.01	1	0.01	ND	ND		
31 alleles			31 alleles		28 alleles			

HLA = human leucocyte antigen; n = number of alleles; RO = Romanian group; HUN = Hungarian group; number of subjects = N (N_1_ = 8333, N_2_ = 1499); number of alleles = 2*N_i_; i =1, 2; AF (%) = relative frequency of alleles (%); ND = not detected; * significant result: *p* < 0.05; *p*-values were obtained from two-proportions z-test; p_BH_ = corrected *p*-values using the Benjamini–Hochberg (BH) method.

**Table 4:** Distribution of HLA-C and DRB1 allele group frequencies in unrelated stem cell donors stratified by ethnicity.

	All Donors (N= 9832)		Ethnic Groups						All Donors (N= 9832)	Ethnic Groups							
			RO (N_1_ = 8333)		HUN (N_2_ = 1499)					RO (N_1_ = 8333)			HUN (N_2_ = 1499)				
HLA-C Alleles	N	AF	n	AF	n	AF	*p*	p_BH_	HLA-DRB1	n	AF	n	AF	n	AF	*p*	p_BH_
*07	4894	24.89	4087	24.52	807	26.92	0.0052 *	0.0146 *	*11	3777	19.21	3289	19.73	488	16.28	<0.0001 *	0.0013 *
*04	3192	16.23	2736	16.42	456	15.21	0.0990	0.1980	*13	2175	11.06	1838	11.03	337	11.24	0.7329	0.8161
*12	2773	14.1	2403	14.42	370	12.34	0.0026 *	0.0091 *	*03	2143	10.9	1788	10.73	355	11.84	0.0719	0.1558
*06	1660	8.44	1392	8.35	268	8.94	0.2872	0.3351	*16	2142	10.89	1870	11.22	272	9.07	0.0005 *	0.0022 *
*02	1519	7.72	1304	7.82	215	7.17	0.2177	0.3351	*07	2116	10.76	1775	10.65	341	11.37	0.2390	0.4439
*03	1303	6.63	1087	6.52	216	7.2	0.1666	0.2916	*01	1926	9.79	1637	9.82	289	9.64	0.7568	0.8161
*01	1091	5.55	965	5.79	126	4.2	0.0005 *	0.0035 *	*15	1753	8.91	1475	8.85	278	9.27	0.4548	0.5912
*15	995	5.06	845	5.07	150	5	0.8778	0.8778	*04	1691	8.6	1400	8.4	291	9.71	0.0189 *	0.0614
*05	693	3.52	564	3.38	129	4.3	0.0120 *	0.0280 *	*14	849	4.32	684	4.1	165	5.5	0.0005 *	0.0022 *
*08	498	2.53	391	2.35	107	3.57	0.0001 *	0.0014 *	*08	410	2.09	342	2.05	68	2.27	0.4458	0.5912
*16	387	1.97	320	1.92	67	2.23	0.2534	0.3351	*12	334	1.7	269	1.61	65	2.17	0.0307 *	0.0798
*14	357	1.82	323	1.94	34	1.13	0.0024 *	0.0091 *	*10	278	1.41	237	1.42	41	1.37	0.8161	0.8161
*17	297	1.51	245	1.47	52	1.73	0.2744	0.3351	*09	70	0.36	62	0.37	8	0.27	0.3734	0.5912
*18	5	0.03	4	0.02	1	0.03	0.7674	0.8264									
14 alleles									13 alleles								

* significant result: *p* < 0.05.

**Table 5:** The most prevalent four-locus haplotypes detected in studied groups described in descending order of their relative frequency (%).

HLA-A-B-C-DRB1														
All Donors (N = 9832)					RO Group (N_1_ = 8333)					HUN Group (N_2_ = 1499)				
A	B	C	DRB1	HF	A	B	C	DRB1	HF	A	B	C	DRB1	HF
*01	*08	*07	*03	4.878	*01	*08	*07	*03	4.788	*01	*08	*07	*03	5.561
*02	*18	*07	*11	2.809	*02	*18	*07	*11	2.965	*02	*18	*07	*11	1.909
*03	*35	*04	*01	1.331	*03	*35	*04	*01	1.385	*24	*35	*04	*11	1.310
*03	*07	*07	*15	0.905	*23	*44	*04	*07	0.936	*03	*07	*07	*15	1.308
*23	*44	*04	*07	0.897	*11	*35	*04	*01	0.875	*02	*13	*06	*07	1.172
*11	*35	*04	*01	0.864	*03	*07	*07	*15	0.836	*03	*35	*04	*01	1.114
*02	*13	*06	*07	0.844	*02	*13	*06	*07	0.811	*02	*27	*02	*16	1.072
*24	*35	*04	*11	0.748	*25	*18	*12	*15	0.764	*01	*40	*15	*14	0.922
*25	*18	*12	*15	0.745	*03	*18	*12	*16	0.742	*11	*35	*04	*01	0.825
*02	*44	*07	*16	0.671	*24	*35	*04	*11	0.715	*02	*07	*07	*15	0.768
*02	*27	*02	*16	0.657	*02	*44	*07	*16	0.662	*02	*40	*03	*13	0.716
*02	*07	*07	*15	0.646	*02	*51	*01	*16	0.636	*02	*44	*07	*16	0.699
*03	*18	*12	*16	0.634	*02	*07	*07	*15	0.632	*23	*44	*04	*07	0.663
*26	*38	*12	*04	0.581	*02	*27	*02	*16	0.611	*24	*07	*07	*15	0.657
*02	*51	*01	*16	0.553	*11	*35	*04	*11	0.584	*02	*08	*07	*03	0.649
*30	*13	*06	*07	0.542	*26	*38	*12	*04	0.578	*25	*18	*12	*15	0.648
*32	*35	*04	*11	0.535	*32	*35	*04	*11	0.570	*30	*13	*06	*07	0.621
*01	*52	*12	*15	0.530	*02	*51	*15	*16	0.561	*02	*56	*06	*07	0.613
*02	*51	*15	*16	0.520	*01	*52	*12	*15	0.554	*02	*38	*12	*13	0.596
*11	*35	*04	*11	0.520	*30	*13	*06	*07	0.543	*26	*38	*12	*04	0.580
*02	*35	*04	*11	0.512	*24	*18	*07	*11	0.528	*01	*56	*06	*07	0.533
*24	*18	*07	*11	0.503	*24	*13	*06	*07	0.504	*02	*35	*04	*11	0.507
*02	*08	*07	*03	0.478	*02	*35	*04	*11	0.4792	*33	*14	*08	*01	0.4953
*01	*40	*15	*14	0.4681	*02	*08	*07	*03	0.4783	*11	*52	*12	*15	0.4876
*24	*13	*06	*07	0.4635	*24	*38	*12	*13	0.4653	*29	*44	*16	*07	0.4623
*24	*38	*12	*13	0.4355	*02	*35	*04	*01	0.4498	*25	*18	*12	*13	0.4397
*66	*41	*17	*13	0.4104	*32	*40	*02	*16	0.4445	*02	*39	*12	*16	0.4237
*33	*14	*08	*01	0.4065	*66	*41	*17	*13	0.4216	*11	*35	*04	*14	0.4109
*32	*40	*02	*16	0.396	*33	*14	*08	*01	0.3912	*02	*35	*04	*14	0.4071
*02	*35	*04	*01	0.3954	*01	*40	*15	*14	0.3861	*31	*40	*03	*04	0.3588
*24	*07	*07	*15	0.3871	*01	*35	*04	*11	0.3739	*02	*41	*17	*13	0.3573
*29	*44	*16	*07	0.3574	*24	*35	*04	*07	0.3735	*03	*35	*04	*11	0.3548
*02	*27	*01	*01	0.3572	*02	*27	*01	*01	0.3614	*02	*44	*05	*01	0.3442
*02	*38	*12	*13	0.3539	*24	*07	*07	*15	0.3581	*02	*27	*01	*01	0.3442
*01	*35	*04	*11	0.3533	*26	*38	*12	*13	0.3524	*02	*44	*05	*04	0.3396
*02	*44	*05	*04	0.3447	*02	*15	*03	*04	0.3505	*02	*52	*12	*15	0.3342
*24	*35	*04	*07	0.3440	*02	*44	*05	*11	0.3490	*69	*41	*17	*13	0.3320
*25	*18	*12	*04	0.3431	*11	*35	*04	*14	0.3473	*01	*52	*12	*15	0.3309
*02	*15	*03	*04	0.3426	*29	*44	*16	*07	0.3382	*11	*35	*04	*07	0.3302
*26	*38	*12	*13	0.3390	*25	*18	*12	*04	0.3297	*68	*14	*08	*13	0.3290
*11	*35	*04	*14	0.3387	*01	*49	*07	*13	0.3284	*03	*07	*07	*16	0.3280
*25	*08	*07	*03	0.3289	*25	*08	*07	*03	0.3257	*24	*18	*07	*11	0.3208
*02	*44	*05	*11	0.3126	*02	*38	*12	*13	0.3170	*24	*08	*07	*03	0.3191
*02	*57	*06	*07	0.3083	*02	*44	*05	*04	0.3121	*02	*51	*15	*04	0.3190
*11	*52	*12	*15	0.3034	*02	*18	*12	*16	0.3121	*03	*35	*04	*04	0.3183
*01	*57	*06	*07	0.2870	*24	*18	*02	*11	0.2994	*02	*44	*05	*15	0.3140
*01	*49	*07	*13	0.2853	*68	*18	*12	*11	0.2980	*26	*38	*12	*13	0.3104
*24	*18	*02	*11	0.2802	*24	*47	*06	*07	0.2887	*02	*40	*03	*04	0.3060
*02	*18	*12	*16	0.2738	*11	*52	*12	*15	0.2746	*01	*08	*07	*11	0.3054
*31	*40	*03	*04	0.2729	*02	*51	*15	*11	0.2733	*23	*44	*04	*11	0.3029
*68	*18	*12	*11	0.2698	*32	*51	*12	*01	0.2723	*26	*08	*07	*03	0.2986
*02	*27	*02	*01	0.2622	*02	*39	*12	*16	0.2691	*25	*18	*12	*04	0.2985
*02	*51	*15	*11	0.2610	*11	*35	*04	*16	0.2674	*23	*49	*07	*11	0.2953
*02	*39	*12	*16	0.2599	*11	*56	*01	*01	0.2669	*11	*35	*04	*04	0.2829
*02	*40	*03	*13	0.2545	*03	*51	*01	*16	0.2641	*02	*14	*08	*01	0.2782
*03	*35	*04	*11	0.2540	*02	*27	*02	*01	0.2638	*02	*27	*02	*04	0.2749
*24	*44	*02	*01	0.2529	*02	*50	*06	*07	0.2623	*24	*40	*02	*11	0.2741
*11	*35	*04	*16	0.2526	*31	*40	*03	*04	0.2579	*03	*07	*07	*11	0.2700
*24	*47	*06	*07	0.2512	*02	*57	*06	*07	0.2570	*02	*39	*12	*01	0.2656
*24	*08	*07	*03	0.2508	*03	*35	*04	*11	0.2552	*01	*44	*02	*16	0.2655
					*24	*44	*02	*01	0.2551	*02	*15	*03	*04	0.2648
					*03	*55	*03	*13	0.2508	*02	*35	*04	*01	0.2618
					*01	*57	*06	*07	0.2506	*11	*18	*07	*11	0.2574
										*02	*49	*07	*11	0.2546
										*24	*35	*04	*04	0.2532

HLA = human leucocyte antigen; RO = Romanian group; HUN = Hungarian group; only haplotypes with frequencies of at least 0.25% were considered; HF (%) = haplotype frequency.

## Data Availability

The raw data presented in this study can be obtained upon reasonable request to Lucia Dican at lucia.dican@umfcluj.ro.

## Supplementary Materials

The following supporting information can be downloaded at https://www.mdpi.com/article/10.3390/life14101243/s1, Table S1. The most prevalent two-locus haplotypes (HLA-A-B and HLA-A-C) detected in two studied groups described in descending order of their relative frequency (%): Tabel S2. The most prevalent two-locus haplotypes (HLA-B-C and HLA-B-DRB1) detected in two studied groups described in descending order of their relative frequency (%): Tabel S3. The most prevalent two-locus haplotypes (HLA-C-DRB1 and HLA-A-DRAB1) detected in two studied groups described in descending order of their relative frequency (%); Table S4. The most prevalent three-locus haplotypes (HLA-A-B-C and HLA-A-B-DRB1) detected in studied groups described in descending order of their relative frequency (%); Tabel S5. The most prevalent three-locus haplotypes (HLA-B-C-DRB1 and HLA-A-C-DRB1) detected in studied groups described in descending order of their relative frequency (%).
