# Supplementary material for: HLA Diversity in Transylvanian Ethnic Groups: Consequences for Hematopoietic Cell Transplantation
Source: Life (Basel). 2024 Sep 28;14(10):1243. doi: 10.3390/life14101243 (PMC11508996; doi:10.3390/life14101243)
Supplement: Supplementary file 1 [file life-14-01243-s001.zip › life-3192431-supplementary.pdf]

Table S1. The most prevalent two-locus haplotypes (HLA-A-B and HLA-A-C) detected in two studied groups described in descending order of their relative frequency (%)

HLA-A-B									HLA-A-C								
All donors (N = 9832)			RO (N_1 = 8333)			HUN (N_2 = 1499)			All donors (N = 9832)			RO (N_1 = 8333)			HUN (N_2 = 1499)		
**A***	**B***	**HF**	**A***	**B***	**HF**	**A***	**B***	**HF**	**A***	**C***	**HF**	**A***	**C***	**HF**	**A***	**C***	**HF**
**01**	**08**	5.887	**01**	**08**	5.709	**01**	**08**	6.882	**02**	**07**	7.578	**02**	**07**	7.656	**01**	**07**	7.981
**02**	**18**	4.095	**02**	**18**	4.288	**02**	**44**	3.964	**01**	**07**	7.116	**01**	**07**	6.947	**02**	**07**	7.247
**02**	**51**	3.430	**02**	**51**	3.507	**02**	**51**	3.015	**03**	**07**	3.158	**03**	**07**	3.039	**03**	**07**	4.090
**02**	**44**	3.379	**02**	**44**	3.284	**02**	**18**	2.993	**02**	**02**	2.781	**02**	**02**	2.809	**02**	**03**	3.126
**11**	**35**	2.674	**11**	**35**	2.735	**03**	**07**	2.714	**11**	**04**	2.675	**11**	**04**	2.740	**02**	**06**	2.916
**03**	**35**	2.588	**03**	**35**	2.624	**03**	**35**	2.537	**03**	**04**	2.635	**03**	**04**	2.598	**02**	**12**	2.767
**24**	**35**	2.348	**24**	**35**	2.336	**11**	**35**	2.367	**02**	**12**	2.536	**02**	**04**	2.517	**03**	**04**	2.745
**03**	**07**	2.244	**02**	**35**	2.195	**24**	**35**	2.366	**02**	**04**	2.509	**02**	**12**	2.502	**02**	**02**	2.682
**02**	**35**	2.163	**03**	**07**	2.151	**02**	**27**	2.077	**02**	**06**	2.325	**02**	**06**	2.240	**24**	**07**	2.598
**02**	**27**	2.068	**02**	**27**	2.052	**02**	**40**	2.052	**24**	**07**	2.224	**24**	**07**	2.115	**01**	**06**	2.466
**02**	**15**	1.664	**02**	**15**	1.709	**25**	**18**	1.994	**02**	**03**	2.143	**24**	**04**	2.016	**24**	**04**	2.434
**25**	**18**	1.623	**25**	**18**	1.559	**02**	**35**	1.845	**24**	**04**	2.062	**26**	**12**	1.975	**11**	**04**	2.308
**02**	**07**	1.563	**26**	**38**	1.547	**02**	**07**	1.745	**01**	**06**	1.988	**02**	**03**	1.946	**02**	**04**	2.294
**26**	**38**	1.506	**02**	**07**	1.531	**01**	**35**	1.655	**26**	**12**	1.925	**24**	**12**	1.914	**25**	**12**	2.246
**01**	**35**	1.431	**03**	**51**	1.497	**02**	**15**	1.490	**02**	**01**	1.804	**01**	**06**	1.905	**02**	**05**	1.893
**03**	**51**	1.424	**01**	**35**	1.393	**24**	**07**	1.361	**24**	**12**	1.790	**02**	**01**	1.899	**02**	**15**	1.715
**02**	**40**	1.399	**24**	**18**	1.361	**02**	**39**	1.357	**25**	**12**	1.760	**03**	**12**	1.806	**26**	**12**	1.660
**24**	**18**	1.329	**23**	**44**	1.313	**23**	**44**	1.331	**03**	**12**	1.642	**25**	**12**	1.665	**23**	**04**	1.413
**23**	**44**	1.315	**02**	**40**	1.270	**26**	**38**	1.285	**02**	**15**	1.628	**02**	**15**	1.634	**01**	**04**	1.390
**32**	**35**	1.204	**32**	**35**	1.269	**24**	**44**	1.198	**02**	**05**	1.579	**02**	**05**	1.520	**30**	**06**	1.225
**24**	**44**	1.172	**03**	**18**	1.192	**02**	**13**	1.196	**01**	**04**	1.448	**01**	**04**	1.471	**24**	**02**	1.221
**02**	**39**	1.144	**24**	**44**	1.166	**24**	**18**	1.183	**23**	**04**	1.444	**24**	**02**	1.452	**02**	**01**	1.218
**03**	**18**	1.106	**02**	**39**	1.135	**01**	**56**	1.125	**24**	**02**	1.409	**23**	**04**	1.451	**01**	**12**	1.176
**02**	**13**	1.055	**24**	**51**	1.056	**30**	**13**	1.119	**24**	**03**	1.205	**24**	**03**	1.269	**11**	**15**	1.159
**24**	**51**	0.980	**02**	**13**	1.030	**01**	**40**	1.062	**32**	**04**	1.173	**32**	**04**	1.237	**24**	**12**	1.064
**24**	**07**	0.961	**24**	**7**	0.894	**11**	**51**	0.996	**01**	**12**	1.083	**24**	**06**	1.112	**11**	**07**	1.050
**30**	**13**	0.896	**30**	**13**	0.857	**03**	**51**	0.983	**24**	**06**	1.033	**01**	**12**	1.064	**11**	**12**	0.995
**11**	**51**	0.857	**11**	**51**	0.826	**03**	**44**	0.973	**11**	**12**	1.003	**32**	**02**	0.997	**02**	**08**	0.976
**01**	**57**	0.817	**68**	**18**	0.808	**02**	**38**	0.909	**30**	**06**	1.000	**11**	**12**	0.994	**01**	**15**	0.940
**02**	**41**	0.771	**24**	**38**	0.777	**33**	**14**	0.890	**32**	**02**	0.954	**30**	**06**	0.958	**26**	**07**	0.861

**03**	**44**	0.760	**32**	**40**	0.776	**02**	**56**	0.853	**11**	**07**	0.842	**11**	**07**	0.814	**03**	**02**	0.852
**68**	**18**	0.740	**02**	**41**	0.769	**32**	**35**	0.831	**11**	**01**	0.752	**32**	**12**	0.813	**33**	**08**	0.844
**33**	**14**	0.729	**01**	**57**	0.762	**11**	**52**	0.828	**01**	**15**	0.740	**11**	**01**	0.811	**24**	**03**	0.825
**24**	**38**	0.716	**01**	**37**	0.733	**02**	**08**	0.813	**24**	**01**	0.736	**24**	**01**	0.805	**32**	**04**	0.798
**02**	**08**	0.706	**03**	**44**	0.719	**24**	**40**	0.785	**03**	**01**	0.731	**03**	**01**	0.742	**03**	**12**	0.767
**32**	**40**	0.702	**01**	**52**	0.713	**02**	**41**	0.773	**32**	**12**	0.718	**03**	**06**	0.716	**29**	**16**	0.760
**01**	**40**	0.701	**33**	**14**	0.700	**29**	**44**	0.752	**33**	**08**	0.705	**01**	**15**	0.699	**02**	**17**	0.747
**01**	**37**	0.698	**02**	**8**	0.689	**03**	**18**	0.686	**29**	**16**	0.690	**33**	**08**	0.679	**32**	**02**	0.720
**29**	**44**	0.697	**29**	**44**	0.683	**24**	**51**	0.676	**26**	**07**	0.687	**29**	**16**	0.677	**03**	**01**	0.702
**01**	**52**	0.689	**32**	**51**	0.659	**02**	**14**	0.673	**03**	**06**	0.686	**02**	**17**	0.671	**68**	**04**	0.667
**24**	**40**	0.665	**24**	**40**	0.651	**01**	**44**	0.653	**03**	**02**	0.675	**68**	**07**	0.670	**03**	**06**	0.667
**11**	**52**	0.618	**24**	**13**	0.647	**02**	**52**	0.603	**02**	**17**	0.674	**26**	**07**	0.659	**23**	**07**	0.648
**32**	**51**	0.616	**01**	**40**	0.632	**68**	**35**	0.552	**11**	**15**	0.625	**03**	**02**	0.629	**32**	**07**	0.622
**24**	**27**	0.604	**24**	**27**	0.622	**68**	**14**	0.525	**68**	**07**	0.624	**03**	**03**	0.615	**24**	**06**	0.603
**24**	**13**	0.593	**31**	**51**	0.605	**01**	**52**	0.511	**03**	**03**	0.602	**02**	**14**	0.597	**02**	**16**	0.527
**31**	**51**	0.560	**01**	**51**	0.578	**01**	**37**	0.506	**02**	**14**	0.591	**68**	**12**	0.588	**24**	**05**	0.508
**02**	**38**	0.544	**11**	**52**	0.572	**03**	**27**	0.505	**32**	**07**	0.567	**33**	**04**	0.582			
**01**	**51**	0.512	**24**	**15**	0.554				**23**	**07**	0.548	**32**	**07**	0.560			
**24**	**15**	0.508	**29**	**7**	0.542				**68**	**12**	0.539	**04**	**15**	0.540			
**66**	**41**	0.500	**33**	**35**	0.536				**33**	**04**	0.505	**31**	**04**	0.534			
			**66**	**41**	0.513				**66**	**17**	0.501	**05**	**07**	0.528			
												**66**	**17**	0.514			
												**24**	**05**	0.507			

HLA = human leucocyte antigen; RO = Romanian group; HUN= Hungarian group; only haplotypes with frequencies of at least 0.5% were considered; HF (%) = haplotype frequency;

Tabel S2. The most prevalent two-locus haplotypes (HLA-B-C and HLA-B-DRB1) detected in two studied groups described in descending order of their relative frequency (%)

**HLA-B-C**									**HLA-B-DRB1**								
**All donors (N = 9832)**			**RO (N_1 = 8333)**			**HUN (N_2 = 1499)**			**All donors (N = 9832)**			**RO (N_1 = 8333)**			**HUN (N_2 = 1499)**		
**B***	**C***	**HF**	**B***	**C***	**HF**	**B***	**C***	**HF**	**B***	**DRB1***	**HF**	**B***	**DRB1***	**HF**	**B***	**DRB1***	**HF**
**35**	**04**	12.949	**35**	**04**	13.165	35	04	11.761	**08**	**03**	6.816	**08**	**03**	6.698	**08**	**03**	7.484
**08**	**07**	8.240	**08**	**07**	8.041	08	07	9.338	**18**	**11**	5.685	**18**	**11**	5.961	**18**	**11**	4.163
**18**	**07**	5.737	**18**	**07**	5.920	07	07	6.507	**35**	**11**	3.799	**35**	**11**	3.907	**07**	**15**	3.429

**01**	**07**	5.229	**07**	**07**	5.015	18	07	4.697	**35**	**01**	3.262	**35**	**01**	3.342	**35**	**11**	3.203
**18**	**12**	3.836	**18**	**12**	3.942	38	12	3.710	**51**	**11**	2.582	**51**	**11**	2.725	**35**	**01**	2.862
**38**	**12**	3.779	**38**	**12**	3.788	44	05	3.566	**07**	**15**	2.526	**51**	**16**	2.448	**13**	**07**	2.517
**13**	**06**	3.362	**51**	**15**	3.354	13	06	3.502	**13**	**07**	2.443	**13**	**07**	2.442	**44**	**07**	2.259
**51**	**15**	3.275	**13**	**06**	3.338	27	02	3.326	**51**	**16**	2.303	**07**	**15**	2.383	**44**	**16**	1.979
**27**	**02**	3.078	**27**	**02**	3.034	18	12	3.223	**44**	**07**	2.041	**44**	**07**	2.000	**51**	**11**	1.688
**44**	**05**	2.902	**44**	**05**	2.779	40	03	2.960	**44**	**11**	1.692	**44**	**11**	1.696	**27**	**16**	1.645
**51**	**01**	2.306	**51**	**01**	2.477	51	15	2.837	**44**	**16**	1.654	**18**	**16**	1.632	**35**	**14**	1.619
**14**	**08**	2.177	**39**	**12**	2.104	14	08	2.769	**38**	**13**	1.594	**38**	**13**	1.603	**38**	**13**	1.610
**52**	**12**	2.103	**52**	**12**	2.103	56	06	2.401	**27**	**01**	1.519	**44**	**16**	1.595	**44**	**11**	1.607
**39**	**12**	2.034	**14**	**08**	2.070	44	04	2.237	**18**	**16**	1.489	**27**	**01**	1.551	**44**	**04**	1.523
**15**	**03**	2.030	**15**	**03**	2.009	15	03	2.149	**52**	**15**	1.385	**52**	**15**	1.391	**40**	**14**	1.489
**40**	**03**	2.018	**44**	**04**	1.894	52	12	2.101	**35**	**14**	1.369	**51**	**04**	1.355	**51**	**16**	1.466
**44**	**04**	1.944	**40**	**02**	1.888	49	07	1.898	**51**	**04**	1.314	**35**	**14**	1.335	**56**	**07**	1.441
**40**	**02**	1.819	**40**	**03**	1.848	41	17	1.701	**27**	**16**	1.160	**51**	**13**	1.194	**52**	**15**	1.357
**49**	**07**	1.729	**51**	**14**	1.814	39	12	1.661	**51**	**13**	1.143	**35**	**13**	1.133	**27**	**01**	1.301
**51**	**14**	1.694	**49**	**07**	1.698	44	07	1.608	**35**	**13**	1.136	**35**	**04**	1.127	**14**	**01**	1.186
**57**	**06**	1.617	**44**	**07**	1.544	44	16	1.465	**35**	**04**	1.096	**27**	**16**	1.079	**40**	**11**	1.107
**44**	**07**	1.559	**57**	**06**	1.476	40	02	1.424	**44**	**04**	1.070	**15**	**04**	1.079	**35**	**13**	1.095
**41**	**17**	1.434	**56**	**03**	1.423	40	15	1.389	**18**	**15**	1.062	**18**	**15**	1.074	**51**	**04**	1.094
**44**	**16**	1.397	**44**	**16**	1.389	51	01	1.336	**15**	**04**	1.006	**35**	**07**	1.009	**40**	**04**	1.028
**55**	**03**	1.357	**41**	**17**	1.386	44	02	1.191	**35**	**07**	0.963	**44**	**04**	0.980	**40**	**13**	1.019
**27**	**01**	1.287	**27**	**01**	1.312	27	01	1.151	**35**	**15**	0.918	**39**	**16**	0.970	**18**	**15**	1.013
**44**	**02**	1.171	**44**	**02**	1.169	51	14	1.025	**39**	**16**	0.912	**35**	**15**	0.963	**41**	**13**	0.995
**37**	**06**	1.108	**37**	**06**	1.146	54	03	1.003	**40**	**11**	0.892	**40**	**16**	0.898	**35**	**04**	0.983
**35**	**12**	1.063	**35**	**12**	1.121	37	06	0.896	**44**	**01**	0.878	**44**	**01**	0.875	**49**	**11**	0.898
**56**	**01**	1.040	**56**	**01**	1.095	39	07	0.854	**14**	**01**	0.873	**40**	**11**	0.866	**38**	**04**	0.887
**50**	**06**	0.997	**50**	**06**	1.032	50	06	0.800	**40**	**16**	0.848	**14**	**01**	0.804	**44**	**01**	0.879
**40**	**15**	0.730	**01**	**15**	0.778	35	12	0.752	**57**	**07**	0.841	**38**	**04**	0.766	**15**	**13**	0.875
**07**	**15**	0.710	**18**	**02**	0.731	55	01	0.734	**41**	**13**	0.802	**35**	**16**	0.760	**51**	**13**	0.832
**51**	**02**	0.681	**51**	**12**	0.726	48	08	0.701	**38**	**04**	0.778	**41**	**13**	0.752	**18**	**16**	0.773
**51**	**12**	0.668	**47**	**06**	0.696	51	02	0.668	**15**	**13**	0.751	**57**	**07**	0.734	**18**	**03**	0.741
**18**	**02**	0.658	**51**	**02**	0.688	15	07	0.571	**35**	**16**	0.741	**15**	**13**	0.729	**35**	**07**	0.733
**58**	**03**	0.651	**58**	**03**	0.680	51	16	0.568	**40**	**14**	0.726	**27**	**11**	0.688	**14**	**13**	0.664
**47**	**06**	0.615	**15**	**04**	0.627	15	04	0.521	**40**	**13**	0.721	**44**	**13**	0.675	**44**	**13**	0.648
**39**	**07**	0.614	**40**	**15**	0.612				**40**	**04**	0.687	**51**	**01**	0.670	**15**	**11**	0.641

**15**	**04**	0.614	**39**	**07**	0.568				**27**	**11**	0.673	**40**	**13**	0.657	**15**	**04**	0.632
**15**	**07**	0.518	**15**	**07**	0.509				**44**	**13**	0.669	**18**	**04**	0.642	**51**	**14**	0.621
									**18**	**04**	0.628	**40**	**04**	0.633	**35**	**16**	0.613
									**51**	**01**	0.613	**40**	**14**	0.600	**39**	**16**	0.608
									**07**	**11**	0.566	**07**	**11**	0.571	**18**	**13**	0.597
									**14**	**07**	0.522	**14**	**07**	0.569	**35**	**15**	0.592
									**15**	**11**	0.516	**07**	**16**	0.540	**18**	**04**	0.592
									**07**	**16**	0.515	**07**	**07**	0.523	**07**	**01**	0.574
									**49**	**11**	0.515	**55**	**13**	0.518	**35**	**03**	0.569
									**35**	**03**	0.513	**49**	**13**	0.514	**15**	**07**	0.560
									**07**	**07**	0.511	**35**	**03**	0.508	**27**	**11**	0.533
															**35**	**12**	0.528
															**39**	**01**	0.502

HLA = human leucocyte antigen; RO = Romanian group; HUN= Hungarian group; only haplotypes with frequencies of at least 0.5% were considered; HF (%) = haplotype frequency;

Tabel S3. The most prevalent two-locus haplotypes (HLA-C-DRB1 and HLA-A-DRAB1) detected in two studied groups described in descending order of their relative frequency (%)

**HLA-C-DRB1**									**HLA-A-DRB1**								
**All donors (N = 9832)**			**RO (N_1 = 8333)**			**HUN (N_2 = 1499)**			**All donors (N = 9832)**			**RO (N_1 = 8333)**			**HUN (N_2 = 1499)**		
**C***	**DRB1***	**HF**	**C***	**DRB1***	**HF**	**C***	**DRB1***	**HF**	**A***	**DRB1***	**HF**	**A***	**DRB1***	**HF**	**A***	**DRB1***	**HF**
**07**	**03**	7.424	**07**	**03**	7.284	**07**	**03**	8.167	**02**	**11**	6.234	**02**	**11**	6.506	**01**	**03**	5.805
**07**	**11**	5.593	**07**	**11**	5.685	**07**	**11**	5.267	**01**	**03**	5.112	**01**	**03**	4.981	**02**	**11**	4.779
**06**	**07**	4.128	**06**	**07**	4.114	**06**	**07**	4.252	**02**	**16**	4.145	**02**	**16**	4.266	**02**	**07**	3.684
**04**	**11**	3.797	**04**	**11**	3.865	**07**	**15**	3.544	**24**	**11**	3.432	**24**	**11**	3.422	**02**	**16**	3.607
**04**	**01**	3.493	**04**	**01**	3.616	**04**	**11**	3.337	**02**	**13**	2.807	**02**	**13**	2.668	**02**	**13**	3.495
**07**	**15**	2.937	**07**	**15**	2.830	**04**	**01**	2.818	**02**	**07**	2.775	**02**	**07**	2.594	**24**	**11**	3.312
**12**	**15**	2.694	**12**	**15**	2.715	**12**	**15**	2.634	**02**	**04**	2.474	**02**	**04**	2.406	**02**	**04**	2.934
**12**	**16**	2.354	**12**	**16**	2.540	**02**	**16**	2.428	**02**	**01**	2.301	**02**	**01**	2.331	**02**	**15**	2.461
**04**	**07**	2.264	**04**	**07**	2.271	**12**	**13**	2.246	**03**	**01**	1.914	**03**	**01**	1.967	**02**	**01**	2.072
**02**	**16**	2.261	**07**	**16**	2.236	**04**	**07**	2.237	**02**	**15**	1.795	**03**	**16**	1.827	**02**	**03**	1.940
**07**	**16**	2.227	**12**	**13**	2.235	**07**	**16**	2.219	**03**	**11**	1.773	**03**	**11**	1.750	**03**	**11**	1.912
**12**	**13**	2.226	**02**	**16**	2.228	**03**	**13**	1.715	**03**	**16**	1.762	**24**	**07**	1.748	**01**	**07**	1.786
**02**	**11**	1.997	**02**	**11**	2.076	**03**	**04**	1.664	**01**	**11**	1.630	**02**	**15**	1.692	**03**	**15**	1.704

**07**	**13**	1.804	**12**	**11**	1.920	**02**	**11**	1.625	**24**	**07**	1.590	**01**	**11**	1.675	**03**	**01**	1.674
**12**	**11**	1.782	**07**	**13**	1.841	**07**	**13**	1.578	**11**	**01**	1.576	**11**	**01**	1.620	**01**	**14**	1.501
**03**	**04**	1.561	**03**	**04**	1.536	**07**	**01**	1.553	**32**	**11**	1.520	**32**	**11**	1.580	**02**	**14**	1.398
**12**	**04**	1.464	**12**	**04**	1.438	**15**	**14**	1.535	**02**	**03**	1.441	**01**	**13**	1.457	**03**	**16**	1.390
**03**	**13**	1.404	**03**	**13**	1.348	**12**	**04**	1.535	**03**	**13**	1.403	**11**	**11**	1.446	**03**	**13**	1.350
**01**	**01**	1.245	**01**	**01**	1.296	**07**	**07**	1.494	**24**	**13**	1.374	**24**	**13**	1.439	**01**	**11**	1.333
**04**	**04**	1.205	**04**	**04**	1.244	**04**	**13**	1.398	**01**	**13**	1.356	**03**	**13**	1.433	**11**	**01**	1.269
**04**	**13**	1.161	**04**	**13**	1.139	**04**	**14**	1.319	**11**	**11**	1.342	**02**	**03**	1.358	**32**	**11**	1.143
**07**	**01**	1.160	**05**	**11**	1.102	**12**	**16**	1.299	**01**	**15**	1.299	**01**	**15**	1.334	**01**	**04**	1.085
**07**	**07**	1.145	**01**	**16**	1.102	**08**	**01**	1.154	**03**	**15**	1.295	**03**	**15**	1.210	**24**	**04**	1.051
**02**	**01**	1.057	**07**	**01**	1.076	**06**	**11**	1.148	**02**	**14**	1.197	**02**	**14**	1.133	**11**	**15**	1.006
**06**	**11**	1.049	**07**	**07**	1.073	**12**	**01**	1.116	**03**	**04**	1.040	**03**	**04**	1.078	**01**	**15**	0.994
**05**	**11**	1.032	**02**	**01**	1.072	**17**	**13**	1.096	**01**	**07**	1.037	**23**	**07**	1.033	**24**	**16**	0.987
**07**	**04**	1.023	**04**	**15**	1.042	**03**	**11**	1.023	**23**	**07**	1.006	**24**	**16**	0.999	**24**	**13**	0.958
**04**	**14**	1.009	**01**	**11**	1.041	**12**	**14**	1.016	**24**	**16**	1.005	**24**	**01**	0.943	**02**	**08**	0.943
**01**	**11**	1.001	**07**	**04**	1.032	**07**	**04**	0.979	**24**	**01**	0.941	**01**	**07**	0.935	**24**	**01**	0.937
**04**	**15**	0.981	**06**	**11**	1.022	**02**	**01**	0.965	**24**	**04**	0.934	**24**	**04**	0.935	**03**	**07**	0.932
**01**	**16**	0.967	**15**	**11**	0.987	**01**	**01**	0.954	**24**	**15**	0.883	**30**	**07**	0.915	**68**	**13**	0.913
**12**	**01**	0.963	**04**	**14**	0.954	**04**	**04**	0.947	**30**	**07**	0.877	**24**	**15**	0.892	**68**	**11**	0.872
**03**	**11**	0.941	**12**	**01**	0.938	**05**	**04**	0.918	**25**	**15**	0.850	**25**	**15**	0.863	**11**	**11**	0.852
**15**	**11**	0.935	**03**	**11**	0.928	**15**	**16**	0.908	**11**	**15**	0.837	**32**	**16**	0.860	**01**	**01**	0.843
**15**	**16**	0.915	**15**	**16**	0.910	**06**	**04**	0.863	**01**	**16**	0.818	**01**	**16**	0.816	**24**	**15**	0.842
**08**	**01**	0.851	**12**	**14**	0.794	**12**	**11**	0.814	**26**	**04**	0.804	**26**	**13**	0.814	**01**	**13**	0.840
**17**	**13**	0.831	**08**	**01**	0.794	**07**	**08**	0.802	**68**	**11**	0.804	**26**	**04**	0.802	**03**	**04**	0.821
**12**	**14**	0.823	**17**	**13**	0.779	**01**	**11**	0.796	**32**	**16**	0.781	**11**	**15**	0.798	**26**	**04**	0.807
**15**	**14**	0.731	**15**	**04**	0.754	**07**	**14**	0.742	**01**	**14**	0.774	**68**	**11**	0.796	**25**	**15**	0.802
**15**	**04**	0.731	**04**	**16**	0.722	**16**	**07**	0.735	**26**	**13**	0.766	**03**	**07**	0.735	**23**	**07**	0.801
**04**	**16**	0.724	**12**	**07**	0.716	**05**	**03**	0.702	**02**	**08**	0.763	**02**	**08**	0.717	**11**	**16**	0.792
**12**	**07**	0.713	**03**	**03**	0.660	**04**	**16**	0.695	**03**	**07**	0.749	**01**	**14**	0.657	**02**	**12**	0.763
**07**	**14**	0.658	**04**	**03**	0.652	**02**	**04**	0.695	**68**	**13**	0.696	**11**	**16**	0.657	**24**	**07**	0.731
**03**	**03**	0.655	**07**	**14**	0.642	**15**	**11**	0.693	**01**	**04**	0.692	**68**	**13**	0.654	**01**	**16**	0.728
**04**	**03**	0.655	**06**	**13**	0.633	**05**	**11**	0.691	**11**	**16**	0.684	**24**	**03**	0.654	**25**	**04**	0.711
**06**	**13**	0.630	**02**	**13**	0.627	**04**	**03**	0.678	**29**	**07**	0.630	**29**	**07**	0.639	**11**	**14**	0.709
**02**	**13**	0.591	**08**	**07**	0.606	**12**	**07**	0.676	**24**	**03**	0.628	**26**	**07**	0.621	**30**	**07**	0.701
**08**	**07**	0.580	**15**	**14**	0.594	**08**	**13**	0.634	**02**	**12**	0.598	**01**	**04**	0.615	**03**	**03**	0.695
**16**	**07**	0.545	**06**	**15**	0.567	**03**	**03**	0.629	**26**	**07**	0.594	**32**	**01**	0.609	**11**	**04**	0.671

**06**	**15**	0.542	**16**	**07**	0.510	**04**	**15**	0.628	**32**	**01**	0.585	**02**	**12**	0.574	**32**	**13**	0.634
**05**	**04**	0.536	**06**	**03**	0.503	**15**	**04**	0.625	**03**	**03**	0.577	**31**	**04**	0.564	**29**	**07**	0.624
**06**	**03**	0.503				**06**	**03**	0.582	**31**	**04**	0.577	**03**	**03**	0.544	**23**	**11**	0.620
						**01**	**13**	0.581	**11**	**07**	0.531	**11**	**07**	0.528	**30**	**03**	0.594
						**06**	**13**	0.555	**11**	**14**	0.511	**24**	**14**	0.506	**31**	**04**	0.575
						**02**	**15**	0.516	**01**	**01**	0.504				**33**	**01**	0.564
						**04**	**08**	0.506							**11**	**13**	0.551
						**03**	**16**	0.506							**11**	**07**	0.547
															**26**	**13**	0.543
															**25**	**07**	0.530
															**24**	**03**	0.518
															**02**	**10**	0.510
															**26**	**03**	0.502

HLA = human leucocyte antigen; RO = Romanian group; HUN= Hungarian group; only haplotypes with frequencies of at least 0.5% were considered; HF (%) = haplotype frequency;

Table S4. The most prevalent three-locus haplotypes (HLA-A-B-C and HLA-A-B-DRB1) detected in studied groups described in descending order of their relative frequency (%)

**HLA-A-B-C**												**HLA-A-B-DRB1**											
**All donors (N = 9832)**				**RO (N_1 = 8333)**				**HUN (N_2 = 1499)**				**All donors (N = 9832)**				**RO (N_1 = 8333)**				**HUN (N_2 = 1499)**			
**A***	**B***	**C***	**HF**	**A***	**B***	**C***	**HF**	**A***	**B***	**C***	**HF**	**A***	**B***	**DRB1***	**HF**	**A***	**B***	**DRB1***	**HF**	**A***	**B***	**DRB1***	**HF**
**01**	**08**	**07**	5.868	**01**	**08**	**07**	5.695	**01**	**08**	**07**	6.831	**01**	**08**	**03**	4.885	**01**	**08**	**03**	4.789	**01**	**08**	**03**	5.576
**02**	**18**	**07**	3.427	**02**	**18**	**07**	3.603	**03**	**07**	**07**	2.683	**02**	**18**	**11**	2.975	**02**	**18**	**11**	3.175	**02**	**18**	**11**	2.010
**11**	**35**	**04**	2.505	**11**	**35**	**04**	2.570	**02**	**18**	**07**	2.678	**03**	**35**	**01**	1.340	**03**	**35**	**01**	1.399	**03**	**07**	**15**	1.262
**03**	**35**	**04**	2.402	**03**	**35**	**04**	2.364	**03**	**35**	**04**	2.577	**02**	**51**	**16**	1.232	**02**	**51**	**16**	1.357	**02**	**13**	**07**	1.222
**03**	**07**	**07**	2.136	**03**	**07**	**07**	2.034	**11**	**35**	**04**	2.287	**24**	**18**	**11**	1.022	**24**	**18**	**11**	1.064	**24**	**35**	**11**	1.184
**24**	**35**	**04**	1.903	**24**	**35**	**04**	1.894	**24**	**35**	**04**	2.109	**02**	**44**	**16**	1.003	**02**	**44**	**16**	1.001	**03**	**35**	**01**	1.114
**02**	**35**	**04**	1.747	**02**	**35**	**04**	1.788	**25**	**18**	**12**	2.014	**24**	**35**	**11**	0.954	**23**	**44**	**07**	0.940	**02**	**44**	**16**	1.052
**25**	**18**	**12**	1.623	**25**	**18**	**12**	1.556	**02**	**04**	**05**	1.840	**03**	**07**	**15**	0.928	**24**	**35**	**11**	0.927	**02**	**07**	**15**	0.945
**26**	**38**	**12**	1.511	**26**	**38**	**12**	1.547	**02**	**27**	**02**	1.635	**23**	**44**	**07**	0.896	**11**	**35**	**01**	0.905	**01**	**40**	**14**	0.905
**02**	**44**	**05**	1.474	**02**	**27**	**02**	1.425	**02**	**35**	**04**	1.560	**11**	**35**	**01**	0.894	**03**	**07**	**15**	0.871	**02**	**27**	**16**	0.901
**02**	**27**	**02**	1.452	**02**	**44**	**05**	1.392	**02**	**40**	**03**	1.460	**02**	**13**	**07**	0.867	**02**	**13**	**07**	0.845	**24**	**18**	**11**	0.862
**02**	**07**	**07**	1.296	**01**	**35**	**04**	1.283	**02**	**07**	**07**	1.439	**02**	**51**	**11**	0.796	**02**	**51**	**11**	0.826	**11**	**35**	**01**	0.845
**23**	**44**	**04**	1.269	**23**	**44**	**04**	1.272	**24**	**07**	**07**	1.306	**25**	**18**	**15**	0.754	**25**	**18**	**15**	0.774	**02**	**51**	**16**	0.802

01	35	04	1.260	02	07	07	1.271	26	38	12	1.302	02	07	15	0.677	03	18	16	0.750	24	07	15	0.749
02	51	15	1.208	02	51	15	1.201	02	13	06	1.293	02	27	01	0.647	02	27	01	0.649	02	40	13	0.734
32	35	04	1.087	32	35	04	1.133	23	44	04	1.264	03	18	16	0.645	02	07	15	0.647	25	18	15	0.638
02	13	06	1.053	02	13	06	1.013	02	51	15	1.194	02	27	16	0.615	32	35	11	0.626	02	08	03	0.637
02	44	07	0.927	02	44	07	0.882	02	44	07	1.112	02	44	11	0.611	02	44	11	0.602	02	51	11	0.625
24	07	07	0.897	02	51	01	0.881	30	13	06	1.103	26	38	04	0.580	02	27	16	0.584	02	56	07	0.620
30	13	06	0.871	02	39	12	0.868	01	35	04	1.067	30	13	07	0.564	26	38	04	0.575	02	44	04	0.615
02	39	12	0.870	03	18	12	0.851	01	56	06	1.029	32	35	11	0.560	11	35	11	0.558	30	13	07	0.613
02	51	01	0.833	30	13	06	0.836	02	39	12	0.972	02	35	11	0.520	30	13	07	0.555	23	44	07	0.607
02	40	03	0.803	24	07	07	0.820	02	08	07	0.938	01	52	15	0.520	01	52	15	0.544	02	38	13	0.576
01	57	06	0.788	24	38	12	0.775	02	15	03	0.932	02	08	03	0.508	03	51	11	0.516	26	38	04	0.570
02	15	03	0.785	32	40	02	0.763	01	40	15	0.910	02	44	04	0.504	02	18	16	0.516	01	56	07	0.564
03	18	12	0.731	02	15	03	0.763	02	56	06	0.865	03	51	11	0.501	68	18	11	0.506	33	14	01	0.552
24	38	12	0.718	01	57	06	0.740	02	38	12	0.864					24	13	07	0.501	01	35	11	0.537
33	14	08	0.712	01	37	06	0.739	33	14	08	0.829									02	27	01	0.536
01	37	06	0.704	01	52	12	0.716	11	51	15	0.752									02	44	11	0.518
32	40	02	0.698	24	18	07	0.703	11	52	12	0.740									02	35	11	0.507
01	52	12	0.694	33	14	08	0.690	29	44	16	0.731												
02	8	07	0.690	02	40	03	0.666	02	41	17	0.689												
24	18	07	0.686	02	08	07	0.665	32	35	04	0.680												
29	44	16	0.673	29	44	16	0.663	02	14	08	0.646												
02	41	17	0.618	24	13	06	0.624	01	37	06	0.552												
11	52	12	0.581	02	41	17	0.619	24	18	07	0.544												
24	13	06	0.571	02	51	14	0.587	02	52	12	0.536												
02	51	14	0.560	11	52	12	0.539	68	14	08	0.532												
02	38	12	0.550	02	18	12	0.538	68	35	04	0.510												
01	40	15	0.505	03	51	01	0.530	02	51	14	0.506												
				33	35	04	0.516	01	52	12	0.503												
				66	41	17	0.503	02	51	01	0.501												

HLA = human leucocyte antigen; RO = Romanian group; HUN= Hungarian group; only haplotypes with frequencies of at least 0.5% were considered; HF (%) = haplotype frequency;

Tabel S5. The most prevalent three-locus haplotypes (HLA-B-C-DRB1 and HLA-A-C-DRB1) detected in studied groups described in descending order of their relative frequency (%)

HLA-B-C-DRB1												HLA-A-C-DRB1											
All donors (N = 9832)				RO (N_1 = 8333)				HU (N_2 = 1499)				All donors (N = 9832)				RO (N_1 = 8333)				HU (N_2 = 1499)			
B*	C*	DRB1*	HF	B*	C*	DRB1*	HF	B*	C*	DRB1*	HF	A*	C*	DRB1*	HF	A*	C*	DRB1*	HF	A*	C*	DRB1*	HF
08	07	03	6.751	08	07	03	6.630	08	07	03	7.444	01	07	03	4.888	01	07	03	4.781	01	07	03	5.609
18	07	11	4.112	18	07	11	4.304	07	07	15	3.111	02	07	11	3.089	02	07	11	3.244	02	06	07	2.051
35	04	11	3.404	35	04	11	3.485	18	07	11	3.104	02	06	07	1.474	02	06	07	1.415	02	07	11	2.017
35	04	01	3.086	35	04	01	3.175	35	04	11	2.905	03	04	01	1.344	03	04	01	1.397	24	04	11	1.325
13	06	07	2.381	13	06	07	2.395	35	04	01	2.700	02	02	16	0.991	02	02	16	1.010	02	07	15	1.093
07	07	15	2.344	07	07	15	2.229	13	06	07	2.376	02	07	16	0.924	02	07	16	0.964	03	07	15	1.093
38	12	13	1.592	38	12	13	1.606	27	02	16	1.708	11	04	01	0.902	23	04	07	0.926	02	07	03	1.056
52	12	15	1.367	52	12	15	1.387	38	12	13	1.599	03	07	15	0.893	11	04	01	0.921	03	04	01	0.997
44	04	07	1.319	44	04	07	1.313	56	06	07	1.389	23	04	07	0.888	03	12	16	0.904	02	02	16	0.982
27	02	16	1.129	18	12	16	1.234	40	15	14	1.353	02	07	15	0.814	03	07	15	0.834	02	03	13	0.974
18	12	16	1.095	51	01	16	1.099	44	04	07	1.275	24	04	11	0.797	24	06	07	0.821	01	15	14	0.896
35	04	13	1.034	35	04	13	1.030	52	12	15	1.248	03	12	16	0.796	25	12	15	0.786	01	06	07	0.844
51	01	16	0.977	27	02	16	1.026	14	08	01	1.142	25	12	15	0.788	02	07	03	0.760	02	07	16	0.813
44	07	16	0.934	39	12	16	0.942	35	04	13	1.102	02	07	03	0.787	02	07	15	0.760	11	04	01	0.787
18	12	15	0.892	44	07	16	0.924	41	17	13	1.061	24	06	07	0.722	01	07	13	0.727	24	07	11	0.781
35	04	14	0.881	35	04	04	0.896	44	07	16	1.047	02	12	16	0.653	24	04	11	0.723	25	12	15	0.755
35	04	04	0.876	18	12	15	0.892	35	04	14	1.011	01	07	13	0.653	02	04	01	0.647	01	07	11	0.739
39	12	16	0.875	18	12	11	0.890	18	12	15	0.933	26	12	04	0.633	02	12	16	0.645	02	04	11	0.705
14	08	01	0.864	44	05	11	0.889	40	03	04	0.904	24	07	11	0.615	26	12	04	0.644	30	06	07	0.673
44	05	11	0.840	35	04	07	0.858	38	12	04	0.901	01	06	07	0.593	01	12	15	0.622	03	07	16	0.673
35	04	07	0.839	35	04	15	0.852	44	05	04	0.842	02	04	11	0.591	24	07	11	0.610	02	12	13	0.666
18	12	11	0.833	35	04	14	0.852	40	03	13	0.837	01	12	15	0.587	11	04	11	0.586	23	04	07	0.636
27	01	01	0.801	15	03	04	0.842	35	04	07	0.822	02	04	01	0.584	02	15	16	0.582	03	07	11	0.624
35	04	15	0.794	27	01	01	0.811	51	15	16	0.781	30	06	07	0.579	32	04	11	0.581	02	07	04	0.606
15	03	04	0.793	14	08	01	0.798	49	07	11	0.776	02	03	04	0.570	02	01	16	0.581	01	06	11	0.604
51	15	16	0.787	51	15	16	0.790	35	04	04	0.730	24	02	11	0.565	24	02	11	0.569	26	12	04	0.577
57	06	07	0.783	51	15	11	0.782	27	01	01	0.712	02	15	16	0.537	02	04	11	0.568	24	02	11	0.563
38	12	04	0.778	38	12	04	0.759	35	04	16	0.710	32	02	16	0.530	32	02	16	0.563	02	04	07	0.560
41	17	13	0.771	40	02	16	0.759	44	16	07	0.676	02	02	11	0.528	30	06	07	0.562	02	12	11	0.558
51	15	11	0.721	35	04	16	0.728	44	05	11	0.672	32	04	11	0.524	01	06	07	0.558	02	07	14	0.547

**35**	**04**	**16**	0.716	**41**	**17**	**13**	0.715	**14**	**08**	**13**	0.663	**11**	**04**	**11**	0.524	**02**	**03**	**04**	0.546	**24**	**07**	**03**	0.529
**40**	**02**	**16**	0.687	**57**	**06**	**07**	0.665	**40**	**02**	**11**	0.624	**02**	**01**	**16**	0.513	**02**	**02**	**11**	0.545				
**40**	**03**	**04**	0.606	**18**	**02**	**11**	0.620	**35**	**04**	**03**	0.620					**24**	**12**	**13**	0.540				
**51**	**15**	**04**	0.600	**51**	**15**	**04**	0.606	**07**	**07**	**01**	0.578					**24**	**12**	**11**	0.519				
**40**	**15**	**14**	0.588	**51**	**01**	**11**	0.588	**39**	**12**	**16**	0.570												
**18**	**02**	**11**	0.573	**07**	**07**	**11**	0.553	**15**	**03**	**04**	0.564												
**07**	**07**	**11**	0.556	**27**	**02**	**01**	0.549	**18**	**12**	**11**	0.547												
**40**	**02**	**11**	0.554	**40**	**03**	**04**	0.546	**07**	**07**	**11**	0.530												
**51**	**01**	**11**	0.549	**14**	**08**	**07**	0.545	**51**	**15**	**04**	0.527												
**35**	**04**	**03**	0.545	**40**	**02**	**11**	0.540	**27**	**02**	**01**	0.526												
**44**	**05**	**04**	0.538	**35**	**04**	**03**	0.539	**44**	**02**	**16**	0.508												
**27**	**02**	**01**	0.538					**15**	**03**	**13**	0.507												
**49**	**07**	**11**	0.513																				
**14**	**08**	**07**	0.507																				

HLA = human leucocyte antigen; RO = Romanian group; HUN= Hungarian group; only haplotypes with frequencies of at least 0.5% were considered; HF (%) = haplotype frequency;
